# Optimization of Vaccination Clinics to Improve Staffing Decisions for COVID-19: A Time-Motion Study

**DOI:** 10.3390/vaccines10122045

**Published:** 2022-11-29

**Authors:** Xinyu Wang, Jinhua Pan, Zhixi Liu, Weibing Wang

**Affiliations:** 1 Department of Epidemiology, School of Public Health, Fudan University, Shanghai 200032, China; 2 Department of Ultrasound Medicine, The First Affiliated Hospital, Zhejiang University School of Medicine, Hangzhou 310003, China; 3 Key Laboratory of Pulsed Power Translational Medicine of Zhejiang Province, Hangzhou 310003, China; 4 Key Lab of Public Health Safety of the Ministry of Education, Shanghai 200032, China

**Keywords:** COVID-19 vaccination, vaccination clinics, personnel allocation, discrete-event simulation model, time-motion

## Abstract

As the COVID-19 pandemic disturbed people’s daily life for more than 2 years, many COVID-19 vaccines have been carried forward systematically to curb the transmission of the virus. However, high vaccination tasks bring great challenges to personnel allocation. We observed nine vaccination clinics in Huzhou and Shanghai and built a discrete-event simulation model to simulate the optimal staffing of vaccination clinics under 10 different scenarios. Based on the result of the simulations, we optimized the allocation of vaccination staff in different stages of epidemic development by province in China. The results showed that optimizing staffing could both boost service utilization and shorten the queuing time for vaccination recipients. Taking Jilin Province as an example, to increase the booster vaccination rate within 3 months, the number of vaccination staff members needed was 2028, with a continuous small-scale breakout and 2,416 under a stable epidemic situation. When there was a shortage of vaccination staff, the total number of vaccination clinic staff members needed could be significantly reduced by combining the preview and registration steps. This study provides theoretical support for the personnel arrangement of COVID-19 vaccinations of a booster dose by province and the assessment of current vaccination staff reserves.

## 1. Introduction

The severe acute respiratory syndrome Coronavirus 2 pandemic has had a direct and strong impact on the global economy, medical care and people’s lives. As of 21 September 2022, it has resulted in over 610 million confirmed cases worldwide and 6,508,521 deaths [1]. Different variants with higher transmissibility are constantly emerging [2,3,4,5,6], such as Delta and Omicron, which are currently the dominant variants. High-coverage vaccination is currently one of the most effective methods to curb this viral transmission [7]. Studies have suggested that the COVID-19 vaccine can provide strong protection to people and effectively reduce the infection rate, hospitalization rate and symptomatic disease rate of COVID-19 [8,9,10,11,12]. Furthermore, antibody titers increased after the third dose of COVID-19 vaccine [13]. Most countries are actively promoting COVID-19 vaccination to achieve herd immunity as soon as possible.

Due to the tense situation of COVID-19, an accelerated vaccination program is crucial to improve the coverage of vaccinated populations, especially for elders. In the early stage of vaccination, people had little information about the COVID-19 vaccine, which led to the low willingness to get vaccinated [14]. Too much staff was available and few people went to get vaccinated. As a result, there was much idle staff. At the later stages of vaccination, however, because of the excellent publicity and popularization of the COVID-19 vaccine, people’s cognitive perception of vaccines was improved and their willingness to get vaccinated gradually grew [15]. The increase of people’s demand for vaccination in a short time put pressure on the vaccine and vaccination staff supplements. Although the vaccination strategy based on repeated booster doses of the original vaccine composition is unlikely to be appropriate or sustainable, the transition from pandemic transmission to endemicity might require booster vaccination for the majority of population because of waning vaccine effectiveness over time, and also to protect against mild and asymptomatic infection with Omicron and Delta variants [16]. With continuous small-scale outbreaks, it is urgent to enhance booster vaccination rate nationwide. Thus, it is necessary to figure out the reasonable arrangement of vaccination staff in different vaccination periods, so as to make effective use of resources and improve the overall vaccination rate as soon as possible.

Discrete-event simulation models were widely used for staffing problems in planning mass vaccinations with antibiotics and vaccines [17,18,19,20]. Most studies have modeled data based on simulations, relying on hypothetical scenarios and expert advice that may be biased for practical reference. Hence, in this study, we constructed a discrete-event simulation model based on on-site data obtained from COVID-19 vaccinations in routine vaccination clinics, makeshift COVID-19 vaccination clinics and routine vaccination in routine vaccination clinics, in order to optimize vaccination staffing for the COVID-19 vaccination. In addition, the results of the simulations were used to explore how to optimize the staffing at different stages of the epidemic development and under different scenarios in each province of China.

## 2. Materials and Methods

### 2.1. Research Sites

Vaccination clinics with different arrival flow were selected by convenience sampling from 2 districts (Songjiang and Minhang) of Shanghai and 4 districts (Nanxun, Anji, Deqing and Changxing) from Huzhou, Zhejiang province. As the conditions were similar at the makeshift vaccination sites in Shanghai, we chose 2 of them to conduct our study. These two cities differed in population and economy levels. Shanghai is a well-developed and highly-connected international city with high population density and a large number of imported cases, whereas Huzhou is a medium-sized city with a lower average income and lower population density.

Overall, a total of 7 routine vaccination clinics in Huzhou and 2 makeshift COVID-19 vaccination clinics in Shanghai were covered. From December 2020 to April 2021, we recorded the vaccination time of both COVID-19 vaccination and routine vaccination in these 9 clinics.

### 2.2. Data Source

The time consumption of each step of the vaccination clinics, the queues and the total time consumption of each vaccination clinic were obtained from cross-sectional and track investigation data collected by a time-motion survey tool.

The number of health care workers and resident population in each province were obtained from the National Bureau of Statistics (http://www.stats.gov.cn/, accessed on 20 April 2022). All health personnel with physician’s licenses and vaccination induction certificates in each province were considered as potential future staff for vaccination. Staff in registration and preview steps in vaccination clinics were relatively less demanding, thus all health personnel in each province were considered as potential future staff in the preview and registration steps (all health personnel including vaccination staff).

The number of COVID-19 vaccination clinics and booster vaccination rates for each province were obtained from the corresponding health council (http://www.nhc.gov.cn/, accessed on 20 April 2022).

### 2.3. Ethical Approval

This study has been approved by the ethics committee of School of Public Health, Fudan university (approval number: IRB# 2020-11-07.58).

### 2.4. Statistical Analysis

The service capability, demand and utilization of each vaccination site were calculated as follows:

Capability = 1/t1,(1)

Demand = 1/t2,(2)

Utilization = Demand/Capability,(3)

Capability was the number of vaccination recipients who could be served at each service desk per hour, whereas t1 referred to the time required to complete the service of each service desk at a time. Demand was the actual number of vaccination recipients who could be served at each service desk per hour, whereas t2 represented the interval time between two adjacent vaccination recipients of each service desk. Utilization represented the actual usage of the staff at each service desk. t1, t2 and t3 were all based on the data from the field survey. The three indicators were calculated by conversion.

### 2.5. Model

#### 2.5.1. The Framework of a Discrete-Event Simulation Model

A simulation model is a numerical evaluation tool that studies various practical methods of system modeling, making assumptions based on various real-world scenarios and constructing models with software for the purpose of system analysis, design and evaluation [21]. To represent the workflow of vaccinations, a discrete-event simulation model was constructed through Arena software and was based on the basic concepts of resources, entities, locations, processes and attributes [21]. In this study, the resources referred to the staff at the preview, registration and vaccination steps, and the entities referred to the vaccination recipients. The location was the service desk where the vaccination staff worked. Processes were routines that connect locations. Attributes were the possible states of resources and entities, such as “available” and “unavailable”. The detailed parameter settings of the attributes of each resource and entity shown in Tables S1 and S2. These elements, combined with the physical layout of the system, allowed for the creation of a computer model to represent the actual operating conditions of the system [21,22].

**Figure 1:**
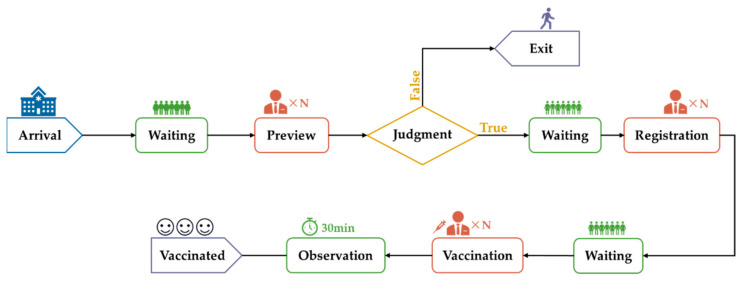
The flow diagram of the vaccination site.

The discrete-event simulation model was constructed using data collected on-site. In this part, vaccination clinics were divided into three sizes: large (daily arrivals 2000–5000), medium (daily arrivals 500–2000) and small (daily arrivals 50–500) according to the flow of arrivals. The basic framework of the discrete-event simulation model was constructed according to the flow diagram of the vaccination site (Figure 1). The vaccination process was completed after the vaccination recipients had gone through preview, registration and vaccination in turn and then waited to be observed for any reactions for 30 min. Taking into account the vaccination contraindications, we set up 99.54% of the vaccinated recipients to meet the requirements for vaccination after a preview step, and the rest of the group left the vaccination clinic.

#### 2.5.2. Parameter Setting

The 8-h vaccination time (8:00–16:00) was set according to the on-site vaccination situation, and the arrival of vaccination recipients in each hour was calculated in equal proportion according to the survey data of the corresponding representative clinic. According to the on-site survey data, the service time of three steps obeyed triangular distribution. The entity represented the vaccination recipients and the resource represented the staff. The way of resource occupation was set as the way of seize-delay-release, i.e., when the entity entered a certain step, the step first allocated the corresponding resource to the entity, and the resource was occupied until the entity released it.

#### 2.5.3. OptQuest Optimization

The OptQuest toolkit enhanced the analysis capabilities of Arena software by automatically finding the optimal solution [23], which was more time efficient and enhanced the accuracy in the controlling variables. In our OptQuest model, the control quantity was the resource to be controlled in the basic model, such as the number of workstations in each step in this model. During each simulation, the value of the control quantity was changed until the optimal value was found and the simulation ended. The constraints were set as the minimum number of vaccination recipients. In this study, the OptQuest toolkit was used to perform simulations for different cases, and the confidence level was set to 95%. The number of simulations was 200 to find the optimal solution for each case. In the OptQuest toolkit, the optimization problem was described and then the control values were found to maximize or minimize the predefined objectives under different optimization objectives.

#### 2.5.4. Scenario Setting

Based on the field data, 10 different scenarios were set up to optimize the vaccination personnel configuration based on the discrete-event simulation model and OptQuest optimization.

We conducted simulations in the two situations, one in which the preview step was retained (Scenarios 1, 3, 5, 7 and 9) and one in which the preview step and the registration step were combined (Scenarios 2, 4, 6, 8 and 10). In each situation, we optimized the staffing that could finally achieve the objectives of the maximum number of vaccination recipients (Scenario 1 and Scenario 2), the least staff number (Scenario 3 and Scenario 4), the highest working efficiency (Scenario 5 and Scenario 6), the least queuing time (Scenario 7 and Scenario 8) and the shortest queue line (Scenario 9 and Scenario 10). In each scenario, the controlled variables were set as the number of workstations in the preview, registration and vaccination steps, and the constraints were the corresponding minimum number of vaccination recipients under different vaccination task conditions with different objectives.

#### 2.5.5. Optimal Staffing for Booster Dose Vaccination at Different Stages

We simulated the optimal vaccination staffing for each province to enhance the booster vaccination rate to 87% (full immunization rate of the COVID-19 vaccine) within 3 months, 6 months, 9 months and 12 months, based on the current booster vaccination condition. Simulations were conducted under two scenarios of continuous small outbreaks and stable condition (no new cases locally), respectively, to assess whether the vaccination staff reserve in each province could meet the vaccination demand. Since the vaccination step had the highest demand for vaccination staff, the optimal allocation of national booster vaccination staff was configured according to the highest efficiency of vaccination step staff in different scenarios.

R 3.6.1 was used for data cleaning and statistical description. Arena software was used to build the discrete-event simulation model and select the optimal personnel arrangement.

## 3. Results

### 3.1. Actual Arrival Situation of Representative Clinics

The representative clinics of large, medium and small vaccination clinics were Shanghai Minhang Gymnasium, the Community Health Service Center of Wukang Street and the Health Center of Hefu Town, respectively. The parameters of the model were set based on the field survey data of these three representative clinics. We found that the number of vaccination recipients arriving at each vaccination clinic reached the peak during 8:00–11:00 and 14:00–15:00, and the number of vaccination recipients arriving during 11:00–12:00 was the least. Figure S1 shows the number of daily arrival rates in three representative vaccination clinics based on field data.

### 3.2. Time Spent in Each Step in Clinics

#### 3.2.1. Basic Information of Vaccination Clinics

A total of nine vaccination clinics and the time required for the five steps of the vaccination process of each clinic were investigated. Nine vaccination clinics and 371 vaccination recipients were observed in the track investigation. In the number taking step, a total of four vaccination clinics and 145 vaccination recipients were investigated. In the preview step, a total of nine vaccination clinics and 710 vaccination recipients were investigated. Eight vaccination clinics and 457 vaccination recipients were investigated in the registration step. A total of 86 vaccination recipients were investigated at two vaccination clinics in the fee charging step, with an average time of 50 s. A total of nine vaccination clinics and 944 vaccination recipients were investigated.

#### 3.2.2. Time Spent in Each Step at Different Vaccination Clinics

Figure S2 and Table S3 show the average time spent by each vaccination recipient during the number taking, preview, registration, charging and vaccination steps at different types of vaccination clinics. We found that makeshift vaccination sites took less time in each step than routine vaccination clinics. Among the five steps of the same vaccination clinic, the registration step and the vaccination step took a longer time than the other steps. For the makeshift vaccination site, COVID-19 vaccination in a routine clinic and routine vaccination in a routine clinic, the registration steps took 25, 115 and115 s, respectively, and the vaccination steps took 82, 155 and 199 s, respectively, whereas the preview and charging steps both needed less than 100 s. Moreover, routine vaccination took longer than COVID-19 vaccinations. In routine clinics, the time spent on each step was similar, whereas the time spent on the vaccination step in makeshift vaccination clinics was much longer than the preview and the registration steps.

#### 3.2.3. Time Spent on the Track Investigation

See Figure S2 and Table S4 for the total time of different kinds of vaccinations at each vaccination clinic. The total vaccination time of the makeshift COVID-19 vaccination sites was relatively short, both less than 300 s. In the same routine clinic, the total time for a routine vaccination was longer than that of COVID-19 vaccination processes. The total time spent on the COVID-19 vaccination process was less than 500 s in all clinics.

### 3.3. Capability, Demand and Utilization of Each Vaccination Clinic

**Figure 2:**
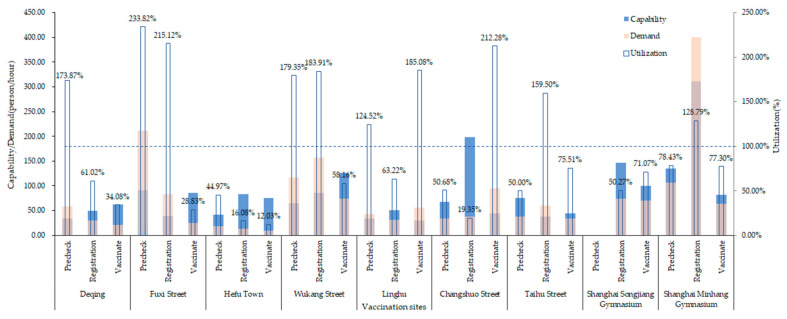
Capability, demand and utilization in each step at each clinic.

Figure 2 shows capability, demand and utilization at different vaccination clinics and different steps calculated by the formula. The utilization rates of each step at the makeshift COVID-19 vaccination clinic were lower than in most routine clinics. In addition, most clinics were overloaded, with utilization rates exceeding 100%.

### 3.4. Queuing Time at Each Clinic

Based on the on-site data, the actual queuing time (including the queuing time for the preview, registration and vaccination steps for each vaccination recipient) of vaccination recipients at each vaccination clinic was calculated (Table S5). Except for Wukang street taking 45 min for queuing, the queuing time of other routine clinics was between 8–15 min, whereas the queuing time at the Shanghai Minhang Gymnasium exceeded one hour, and the queuing time at the Shanghai Songjiang Gymnasium, which was on a similar scale as the Minhang Gymnasium, was only 4 min and 23 s. The difference between the two vaccination clinics was that Shanghai Minhang Gymnasium had set up a preview step, whereas the preview and registration steps were combined at the Shanghai Songjiang Gymnasium. By removing the queuing time for the preview step from total queuing time at the vaccination clinics, it was found that the queuing time at the Shanghai Minhang Gymnasium was significantly reduced, and the queuing time at other vaccination clinics was also reduced to varying degrees.

### 3.5. Optimal Staffing under Different Scenarios

#### 3.5.1. Scenarios 1 and 2

Table 1 shows the optimal staffing that can accomplish the highest number of vaccination recipients given a certain number of required vaccination tasks. In a small vaccination clinic, for example, if the number of daily arrivals was about 100, the optimal configuration was one staff member at the preview step, one staff member at the registration step and two staff members at the vaccination step; if the preview and registration steps were combined, the optimal arrangement was to arrange for one registration staff member and two vaccination staff members. At a medium-sized vaccination clinic, if the number of daily arrivals was about 1000, the optimal staffing was five preview staff members, four registration staff members and six vaccination staff members, for a total of 15 staff members. After combining the preview and registration steps, the optimal staffing was four registration staff members and seven vaccination staff members, requiring only 11 staff members and reducing the staffing by four members. In large vaccination clinics, such as those with a daily arrival of about 3000 vaccination recipients, the optimal staffing was nine preview staff members, eight registration staff members and 13 vaccination staff members, requiring a total of 30 staff members, whereas after combining the preview and registration steps, the optimal staffing was eight registration staff members and 16 vaccination staff members, requiring a total of 24 staff members, reducing staffing by six compared to the three steps. The staff efficiency increased while completing the same amount of vaccination tasks (see Table S6 for detailed staffing). In the case of requiring the largest number of vaccination recipients, for every 500 additional daily arrivals at the vaccination clinic, one to three additional preview staff members, one to two registration staff members and one to three vaccination staff were required, and the number of preview staff could be reduced accordingly after combining the preview and registration steps.

#### 3.5.2. Scenarios 3–6

Table 2 shows the optimal staffing required for a given vaccination task under the objectives of the minimum number of staff and the highest efficiency. In the case of requiring the least number of staff, for every 500 additional daily arrivals at the vaccination clinic, one to two additional preview staff members, one to two registration staff members and one to three vaccination staff members were required. In the case of requiring the highest efficiency, for every 500 additional daily arrivals at the vaccination clinic, one to two additional preview staff members, one to two registration staff members and one to three vaccination staff members were required, and the number of preview staff members could be reduced accordingly after combining the preview and registration steps. See Table S7 for detailed staffing under scenario 3–6.

#### 3.5.3. Scenarios 7–10

Table 3 shows the optimal staffing configuration that required the least queuing time and the shortest queuing line for a certain number of vaccination tasks. In the case of vaccinating the same number of vaccine recipients, the staff requirement was reduced and the work efficiency was increased (Table 3). The queuing time was less than 20 min for all vaccination clinics with 4500 or fewer arrivals (Table S8), which was significantly shorter than the queuing time at the actual observed vaccination clinics, and after combining preview and registration, the queuing time was only 13.85 min even for a large vaccination clinic with 5000 daily arrivals (Table S8). Compared with the queuing time at the actual vaccination clinic (Table S5), the queuing time at the vaccination clinic with the preview step was significantly shorter with optimal staffing (Table 3), and the queuing time at the vaccination clinic after combining the preview and the registration steps was further reduced with the optimal configuration of preview (Table 3). Under the condition that the vaccination queuing time was minimized when a certain number of vaccination tasks were required, one to three additional preview staff members, one to two registration staff members and one to three vaccination staff were required for each additional 500 daily arrivals at the vaccination clinic, and the number of staff members for the preview step could be reduced accordingly after merging the preview and registration steps. In the case of vaccinating the same vaccination target, fewer staff were required, and accordingly staff efficiency increased (see Table S8 for detailed staffing). To achieve the shortest queuing line, for every 500 additional vaccination recipients arriving at the vaccination clinic daily, one to two preview staff members, one to two registration staff members and one to three vaccination staff members were needed.

### 3.6. Capacity Assessment of Optimal Staffing

With the two scenarios of retaining the preview step and combined preview and the registration step, the service capacity was calculated by Formula (4) for the optimal staffing scenarios with different study objectives.

Service capacity = Vaccination recipients/Arrival rate(4)

Except for the service capacity of vaccination clinics with less than 100 arrivals, which was less than 80%, the service capacity of vaccination clinics with different numbers of vaccination recipients (representing the size of the vaccination clinics) were higher, basically around 100% (Table 4). Moreover, the service capacity of our simulations was higher than the actual observed service capacity and efficiency of the current vaccination clinics, and the optimal staffing was reasonable.

### 3.7. Optimization of Staffing for Booster Vaccination in China

#### 3.7.1. Booster Vaccination Human Resource Situation and Progress Nationwide

For provinces with higher population densities, such as Shandong, Henan and Guangdong provinces, there were correspondingly more healthcare workers available to serve as vaccination clinic staff. However, for provinces where the current epidemic was more severe, such as Jilin and Fujian, there were relatively fewer staff, and there was a need to speed up the booster vaccination and also to ensure front-line vaccination staff. Thus, it is necessary to optimize the allocation of vaccination personnel in different provinces for different stages of the current epidemic’s development, so as to improve service efficiency and utilization and to obtain more staff members for front-line vaccination (Figure S3).

Based on the collected data, it was found that the highest booster vaccination rate was in Beijing with 54.8%, followed by Shandong Province, Shanghai and Tianjin, all with booster vaccination rates above 40%. The lower booster vaccination rates were found in Shanxi and Hebei provinces (Figure S4). With small-scale outbreaks occurring across the country, provinces needed to accelerate the pace of booster vaccination.

#### 3.7.2. Optimization of Staffing for Vaccination When Small-Scale Outbreaks Continued to Occur

When small-scale outbreaks were constantly occurring, in order to ensure available front-line epidemic prevention staff members, as well as to achieve the need for further vaccination, scenarios 3, 4, 5 and 6 could be referred to when optimizing vaccination personnel allocation, i.e., under the premise that the vaccination volume task was certain. Additionally, the simulation was conducted under two situations, one in which the clinics were equipped with preview step and the other in which the preview and registration steps were combined. Since the vaccination tasks in each province were well beyond the scope of our simulation, we used the results of the maximum number of arrivals (5000) for the optimal staffing simulation for provinces under different scenarios. The optimal vaccination staffing for each province with the objective of the least number of staff members and the highest efficiency was studied, assuming that the provinces increased the booster vaccination rate to the level of the full vaccination rate (87%) at 3, 6, 9 and 12 months when small-scale outbreaks were constantly occurring.

**Figure 3:**
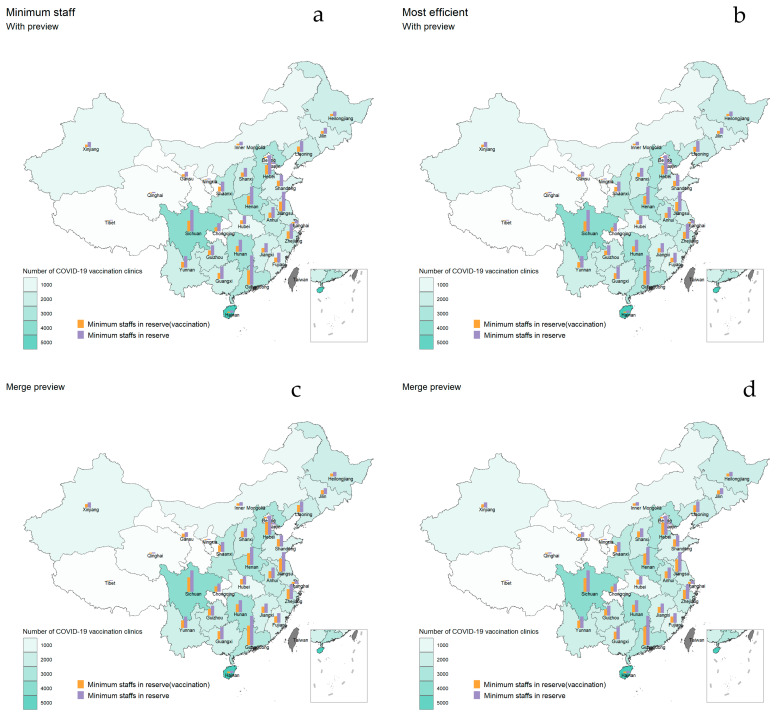
Optimal staffing to achieve the goals within 3 months. The green area in the map represents the number of COVID-19 vaccination clinics in each province, and the darker the color represents more COVID-19 vaccination clinics. The orange bar represents the minimum number of staff members needed to be stockpiled for the vaccination step at the vaccination clinics optimized by the simulation model under the goal of raising the booster vaccination rate to the full immunization level in 3 months in different scenarios. The higher the bar, the more healthcare workers needed to be stocked. The purple bar represents the minimum number of staff members to be stocked in the vaccination clinics, and the higher the bar, the higher the number of staff members are needed to be stocked. (**a**,**b**) Optimization when the vaccination clinic was equipped with preview, registration and vaccination steps; (**c**,**d**) Optimization when the preview and registration steps were combined.

The highest number of COVID-19 vaccination clinics in each province nationwide was in Hainan Province, followed by Sichuan Province, Hunan Province and Hebei Province, all with more than 3000 vaccination clinics, whereas Xinjiang had the lowest number of vaccination clinics, followed by Tibet and Gansu Province (except for four municipalities), all with less than 400 vaccination clinics. Assuming that each province was required to increase the booster vaccination rate to the full immunization level within 3 months in the event of a continuous small-scale outbreak, the optimal staffing for each province is shown in Figure 3. In early 2022, the province with the highest number of new confirmed cases per day was Jilin Province, which required a minimum staffing of 992 vaccination step staff members. A total of 268,933 healthcare workers in the province were certified as inoculation induction and occupational physicians, and the number of vaccination personnel needed for optimization accounted for 0.37% of the number of all staff available as vaccination step workers. In addition to requiring staff efficiency, a minimum reserve of 949 vaccination step staff members was required, representing 0.35% of the total number of staff available as vaccination step staff. If the preview and registration steps were combined, the corresponding number of vaccination staff also decreased, and the optimal staffing could be reduced by 43 staff members under the scenario requiring the least number of staff members, and by 28 staff members under the scenario requiring the highest efficiency. The total number of staff in the vaccination clinics, in the scenario with the preview step and minimum staffing requirements, the optimal staffing required a minimum of 2028 healthcare workers in Jilin Province, and there was 401,501 health personnel in Jilin Province. After combining the preview and registration steps, the optimal staffing required a minimum of 1424 staff members in the province, accounting for 0.35% of the total number of all those available. When staff efficiency was required to be at its highest, the optimal allocation was a minimum of 2028 vaccination clinic staff members in the province, and after combining preview and registration steps, 1454 staff members were required, and the province could reduce the number of vaccination staff members by 574. The optimal staffing for each province to increase booster vaccination rates to full immunization levels within 6 months is shown in Figure S5.

#### 3.7.3. Optimal Allocation of Vaccination Personnel for Booster When the Epidemic Was Stable

**Figure 4:**
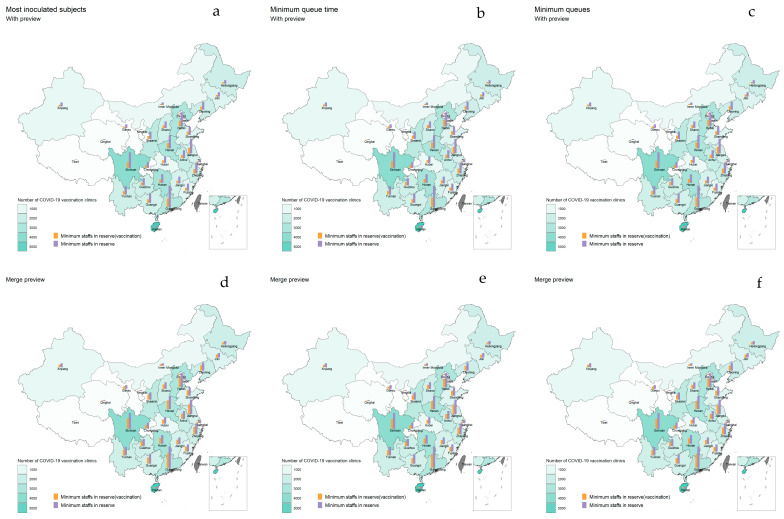
Optimal staffing to achieve the goals within 3 months when the epidemic was stable. The green area in the map represents the number of COVID-19 vaccination clinics in each province, and the darker the color, the more COVID-19 vaccination clinics there are. The orange bar represents the minimum number of staff needed to be stockpiled for the vaccination step at the vaccination clinics optimized by the simulation model under the goal of raising the booster vaccination rate to the full immunization level in 3 months in different scenarios. The higher the bar, the more healthcare workers are needed to be stocked. The purple bar represents the minimum number of staff members to be stocked in the vaccination clinics, and the higher the bar, the higher the number of staff members to be stocked. (**a**–**c**) Optimization when the vaccination clinic was equipped with preview, registration and vaccination steps; (**d**–**f**) Optimization when the preview and registration steps were combined.

In this study, epidemic stability was defined as a situation where there are no small-scale outbreaks and no new indigenous cases. At this point, scenarios 1, 2, 7, 8, 9 and 10 could be referred to as having optimizing vaccination personnel allocation. The simulation study was conducted to optimize staff allocation based on the objectives of vaccinating the most vaccinated recipients, the least queuing time and the shortest vaccination queues, respectively. Furthermore, the simulation was conducted under two situations, one in which the clinics were equipped with the preview step and one in which the preview and registration steps were combined. The study assumed optimal vaccination personnel allocation for each province when the epidemic was stable and the provinces increased the vaccination rate of booster shots to the level of the full vaccination rate (87%) within 3, 6, 9 and 12 months. The optimal staffing for each province to increase booster vaccination rates to full immunization levels within 3 months and 6 months are shown in Figure 4 and Figure S6, respectively.

## 4. Discussion

Based on the time-motion survey tool and the time spent by vaccination recipients on each step of the clinic and throughout the whole vaccination process, we constructed a discrete-event simulation model using real-world parameters and set up 10 different scenarios to optimize the vaccination personnel allocation. According to our study, there was still some irrationality in the staffing arrangement of routine vaccination clinics during the initial period of COVID-19 vaccinations, including low staff utilization and overstaffing in some vaccination clinics, whereas the workload of staff in some vaccination clinics was overloaded (utilization rate > 100%). Both overstaffing and understaffing indicated the need to optimize the staffing of vaccination clinics. In one of the survey sites, the Fuxi Street Health Service Center, for example, the demand for its preview and registration steps was much greater than its capability, whereas the utilization rate of the vaccination step was only 29%. This situation suggested that the overload in the first two steps was likely caused by the sparse staffing in the preview step or registration step, resulting in the congestion of vaccination recipients in the first two steps. According to the simulation results of our model, the goal of optimizing staff utilization at each step can be achieved by maintaining the original number of vaccination step staff and setting up two staff members in each of the preview and registration sessions, provided that the vaccination demand of that clinic (n = 250) is met. Survey data from China showed that vaccination staff considered the number of vaccinations in a day at 30 as their fatigue threshold [24,25], but their actual workload was much higher. In addition, the number of staff members was unevenly distributed across regions, with some regions overloaded [24,26,27]. Therefore, the number of staff members at each vaccination clinic and vaccination step should be reasonably set to improve staff job satisfaction and reduce the queuing time of vaccination recipients.

It was found that based on the optimal results, the service utilization and service efficiency of vaccination clinics were significantly improved and the queuing time of vaccination recipients was reduced accordingly. Therefore, we used the results of the model to simulate the optimal vaccination personnel allocation in various provinces of China under different scenarios when small-scale epidemics occurred continuously and after the epidemic was stabilized. In Jilin province, for example, if the booster vaccination rate was required to reach the full vaccination rate within 3 months when small-scale outbreaks were occurring, the optimal staffing was a minimum of 2028 staff members at vaccination clinics across the province. If the target was required to be met within 6 months, the corresponding number was halved. If 9 months and 12 months were required, the corresponding number was reduced by 67% and 75%, respectively. When the epidemic situation was stable and there were no more new native cases on a daily basis, with the objectives of the shortest queuing time and the highest number of vaccination recipients, 2,416 vaccination clinic staff members could be arranged in Jilin Province in order to complete the target within 3 months. The other needed healthcare workers within different requested times were reduced in the same manner as the previous situation. When there was a shortage of vaccination staff, the total number of staff members required could be significantly reduced by combining the preview and registration steps.

When the number of daily arrivals is consistent, the scenario with the smallest staffing will occur in the scenario that requires the least number of staff members to be scheduled and the scenario that requires the highest efficiency. Therefore, our study assumed that the optimal staffing of vaccination clinics would be carried out in these two scenarios in each province when there were continuous small-scale outbreaks in China. The scenarios that required the greatest staffing and the highest number of vaccination recipients would occur in the scenario that required the shortest queuing time, the fewest people queuing and the maximum of vaccination recipients; hence, the study hypothesized that provinces would optimize vaccination personnel allocation in these three scenarios to achieve vaccination goals after the epidemic stabilized. It was found that regardless of the scenario, Guangdong province had the highest optimal allocation of staffing and total number of staff members in the vaccination step, which may be due to the larger resident population in Guangdong province, and may also be due to the earlier data collected in this study on the booster vaccination rate in Guangdong province, resulting in a heavier task to complete the 87% vaccination rate. Each province could optimize vaccination staffing for different situations and goals based on the actual real-time booster vaccination rate in the province, combined with the detailed table of optimal personnel allocation for different scenarios in this study. In some of the optimal staffing, the number of staff required for the vaccination step in the vaccination clinics also decreased after the merger of the preview and registration steps, which may be due to the fact that the time required for the registration step was extended after the merger of the preview and registration steps, resulting in fewer vaccination recipients entering the vaccination step at the same time, and thus creating a corresponding decrease in the number of staff required for the vaccination step. Due to the better organization level and compliance of adults, the technical specification for vaccination against novel coronavirus in China recommends that the number of vaccination recipients per vaccination table and per vaccination worker per hour of vaccination services in group vaccination of adults can be 20–30 [28], and the model measurements of this study were all within this range, i.e., the workload arrangement of vaccination workers was relatively reasonable.

Studies on vaccination clinic personnel in China mainly focus on the current situation of vaccination personnel allocation and vaccination satisfaction, assessment of the service capability and status of vaccination clinics. Fewer studies focus on the optimal allocation of vaccination clinic personnel. For the studies about optimal allocation of vaccination personnel, Hupert [29] used a discrete-event simulation model to study staffing levels for entry screening, triage, medical assessment and drug distribution stations based on a hypothetical antibiotic distribution center under low, medium and high bioterrorism risk response scenarios for bioterrorism threats, and ultimately concluded that the discrete-event simulation model was optimized for higher average staff utilization and reasonable staffing optimization. Beeler [30] used a discrete-event simulation model to study staffing during mass vaccination with influenza vaccines, which focused on the impact of staffing levels on patient queuing times, operational costs and risk of influenza transmission within outpatient clinics, but did not give a specific optimal staffing for influenza vaccines. Andress [19] and Phillips and Williamson [20] discussed some insights on mass vaccination management generated from actual mass vaccination operations, but did not specifically discuss how to optimize staffing decisions. All of the above results were based on hypothetical scenarios, the model parameters were not the real parameters of the study and the optimal allocation of vaccination personnel for COVID-19 vaccinations was not reported. In contrast, our study was based on the real survey data in the case of COVID-19 vaccinations and other routine vaccinations, which is more instructive for practical work. Our study can also provide reference for the personnel arrangement of subsequent COVID-19 vaccine booster dose vaccinations, routine vaccinations or other vaccination work.

## 5. Conclusions

In conjunction with the on-site situation, the staffing in the provinces can be optimized with reference to the results of the simulations, in order to prepare the appropriate staff reserve and improve efficiency. In view of the fact that the booster vaccination rate in China is not yet high and the epidemic is severe, and under the condition of limited medical and health resources, it is necessary to protect the front-line medical resources on the one hand and speed up the booster vaccination on the other hand. The staffing of the provinces can be optimized with reference to the scenario with the least number of staff and the highest efficiency. If the epidemic situation is stabilized, the vaccination staffing in different provinces can be optimized with reference to the scenarios with the most vaccinated recipients, the shortest queuing time and the least number of vaccination recipients in the queue. To complete the vaccination task of 100 vaccination recipients, three to four staff members are required. For every 500 additional daily arrivals at the vaccination clinic, one to three additional preview staff members, one to two registration staff members and one to three vaccination staff members are required. In the case of large-scale emergency vaccinations, the preview step can also be combined with the registration step to improve service efficiency and reduce queuing times and staffing arrangements.

## Figures and Tables

**Table 1:** Optimal staffing under the target of maximum number of vaccination recipients.

Arrival Rate	With Preview Step ^1^					Without Preview Step ^2^			
	Vaccination Recipients	Preview	Registration	Vaccination	Total Staff	Vaccination Recipients	Registration	Vaccination	Total Staff
105	104	1	1	2	4	94	1	2	3
1009	945	5	4	6	15	947	4	7	11
3007	3077	9	8	13	30	3044	8	16	24

^1^ With preview step: the vaccination clinic was equipped with preview, registration and vaccination steps. ^2^ Without preview step: the vaccination clinic was equipped with two steps, whereas the preview and registration steps were combined.

**Table 2:** Optimal staffing under the target of minimum number of staff and highest efficiency.

Arrival Rate	Minimum Staff Number									Highest Efficiency								
	With Preview Step ^1^					Without Preview Step ^2^				With Preview Step ^1^					Without Preview Step ^2^			
	Vaccinated	Preview	Registration	Vaccination	Total Staff	Vaccinated	Registration	Vaccination	Total Staff	Vaccinated	Preview	Registration	Vaccination	Total Staff	Vaccinated	Registration	Vaccination	Total Staff
105	104	1	1	1	3	94	1	1	2	104	1	1	1	3	94	1	1	2
1009	920	4	4	6	14	936	4	6	10	935	4	4	6	14	936	4	6	10
3007	2901	8	8	16	32	2929	8	15	23	2901	9	8	14	31	2911	8	13	21

^1^ With preview step: the vaccination clinic was equipped with preview, registration and vaccination steps. ^2^ Without preview step: the vaccination clinic was equipped with two steps, whereas the preview and registration steps were combined.

**Table 3:** Optimal staffing under the target of least queuing time and shortest queuing number.

Arrival Rate	Minimum Queuing Time									Minimum Queuing Number								
	With Preview Step ^1^					Without Preview Step ^2^				With Preview Step ^1^					Without Preview Step ^2^			
	Queuing Time	Preview	Registration	Vaccination	Total Staff	Queuing time	Registration	Vaccination	Total Staff	Queuing Number	Preview	Registration	Vaccination	Total Staff	Queuing number	Registration	Vaccination	Total Staff
105	0	1	1	2	4	0	1	2	3	0	1	1	2	4	0	1	2	3
1009	0.24	4	4	10	18	0.06	4	10	14	0	4	4	10	18	0	4	10	14
3007	0.24	8	10	18	36	0.42	10	18	28	1	9	10	18	37	3	9	18	27

^1^ With preview step: the vaccination clinic was equipped with preview, registration and vaccination steps. ^2^ Without preview step: the vaccination clinic was equipped with two steps, whereas the preview and registration steps were combined.

**Table 4:** Service utilization for optimal staffing for each purpose.

Arrival Rate	With Preview Step ^1^						Without Preview Step ^2^					
	Maximum Vaccination Recipients		Minimum Staff Number		Highest Efficiency		Maximum Vaccination Recipients		Minimum Staff Number		Highest Efficiency	
	Vaccination Recipients	Service Capacity	Vaccination Recipients	Service Capacity	Vaccination Recipients	Service Capacity	Vaccination Recipients	Service Capacity	Vaccination Recipients	Service Capacity	Vaccination Recipients	Service Capacity
105	104	1	104	1	104	1	94	0.9	94	0.9	94	0.9
1009	945	0.94	920	0.92	935	0.93	947	0.94	936	0.93	936	0.93
3007	3077	1.03	2901	0.97	2901	0.97	3044	1.02	2929	0.98	2911	0.97

^1^ With preview step: the vaccination clinic was equipped with preview, registration and vaccination steps. ^2^ Without preview step: the vaccination clinic was equipped with two steps, whereas the preview and registration steps were combined.

## Data Availability

The data presented in this study are available on request from the corresponding author.

## Supplementary Materials

The following supporting information can be downloaded at: https://www.mdpi.com/article/10.3390/vaccines10122045/s1, Figure S1: Arrival rate of representative clinics; Figure S2: Time spent on each step in each clinic; Figure S3: Distribution of the number of population and staff by province; Figure S4: Booster vaccination rate by province; Figure S5: Optimal staffing to achieve the goals within 6 months; Figure S6: Optimal staffing to achieve the goals within 3 months when the epidemic was stable; Table S1: Parameter settings of resource and entity; Table S2: Other parameter settings of the basic model; Table S3: Time spent on different steps at different clinics; Table S4: Time spent on the whole process at different clinics; Table S5: Queuing time at the actual vaccination clinics; Table S6: Optimal staffing under the target of maximum number of vaccination recipients; Table S7: Optimal staffing under the target of minimum number of staff and highest efficiency; Table S8: Optimal staffing under the target of least queuing time and shortest queuing number; Table S9: Service utilization for optimal staffing for each purpose.
