# Supplementary material for: Optimization of Vaccination Clinics to Improve Staffing Decisions for COVID-19: A Time-Motion Study
Source: Vaccines (Basel). 2022 Nov 29;10(12):2045. doi: 10.3390/vaccines10122045 (PMC9781296; doi:10.3390/vaccines10122045)
Supplement: Supplementary file 1 [file vaccines-10-02045-s001.zip › vaccines-1989333-supplementary.pdf]

vaccines

Manuscript ID: vaccines-1989333

Title: Optimization of Vaccination Clinics to Improve Staffing Decisions for COVID-19: A Time Motion study

Table S1. Parameter settings of resource and entity

	Data Distribution Type	Minimun (min)	Average (min)	Maximum (min)	Queuing time (min)
**Large**					
Preview	Triangle	0.10	0.44	1.50	0.57
Registration	Triangle	0.08	0.39	2.00	0.15
Vaccination	Triangle	2.82	3.77	5.35	0.95
**Medium**					
Preview	Triangle	0.82	1.62	2.78	0.52
Registration	Triangle	1.02	2.19	3.73	0.38
Vaccination	Triangle	0.70	2.18	3.90	0.82
**Small**					
Preview	Triangle	0.15	2.39	3.95	3.23
Registration	Triangle	0.60	0.99	1.98	4.47
Vaccination	Triangle	0.63	1.59	2.68	6.60

Table S2. Other parameter settings of the basic model

Content	Value
Working time	8:00-16:00
Inoculable ratio	99.54%
Number of staff per workstation	1
Observation time (min)	30

Table S3. Time spent in different steps of different clinics

Street	Sample size	Average time (s)	Standard deviation
**Number taking**			
Hefu Town	5	42.6	33.3
Taihu Street	56	44.2	59.5
Changshuo Street	75	63.2	73.1
Linghu	9	141.8	141.6
**Preview**			
Changshuo Street	88	40.9	19.4
Deqing	62	107.8	48.1
Fuxi Street	26	93.4	46.2

Hefu Town	36	174.5	61.8
Linghu	73	104.6	61.3
Taihu Street	67	88.3	109.2
Wukang Street	49	111.2	46.4
Shanghai Minhang Gymnasium	145	26.8	19.9
**Registration**			
Changshuo Street (Routine vaccination)	30	261.0	883.4
Changshuo Street (COVID-19 vaccination)	72	54.6	28.0
Deqing (Routine vaccination)	38	144.0	90.2
Fuxi Street (COVID-19 vaccination)	21	92.5	34.3
Hefu Town (COVID-19 vaccination)	35	86.2	58.6
Linghu (COVID-19 vaccination)	46	214.3	89.1
Taihu Street (Routine vaccination)	52	92.3	80.1
Taihu Street (COVID-19 vaccination)	17	191.4	87.2
Wukang Street (Routine vaccination)	37	169.2	82.0
Shanghai Minhang Gymnasium	109	26.2	13.8
Shanghai Songjiang Gymnasium	174	24.6	26.3
**Charging**			
Changshuo Street	52	51.5	18.6
Wukang Street	34	48.6	15.2
**Vaccination**			
Changshuo Street (Routine vaccination)	26	242.0	70.2

Changshuo Street (COVID-19 vaccination)	56	83.4	25.1
Deqing (Routine vaccination)	55	230.4	92.8
Fuxi Street (COVID-19 vaccination)	23	83.6	21.3
Hefu Town (COVID-19 vaccination)	43	95.3	22.4
Linghu (Routine vaccination)	107	236.9	107.1
Taihu Street (COVID-19 vaccination)	60	163.1	64.3
Wukang Street (Routine vaccination)	123	142.5	61.3
Shanghai Minhang Gymnasium	282	88.1	24.0
Shanghai Songjiang Gymnasium	169	72.5	27.5

Table S4. Time spent of whole process in different clinics

	**Sample size**	**Average time (s)**	**Standard deviation**
Changshuo Street (Routine vaccination)	14	1122.4	340.8
Changshuo Street (COVID-19 vaccination)	31	255.6	112.2
Deqing (Routine vaccination)	25	769.2	268.3
Fuxi Street (COVID-19 vaccination)	22	336.0	64.5
Hefu Town (Routine vaccination)	2	839.0	39.6
Hefu Town (COVID-19 vaccination)	39	445.4	146.5
Linghu (Routine vaccination)	50	1015.5	380.7
Taihu Street (COVID-19 vaccination)	37	404.2	235.8
Wukang Street (Routine vaccination)	21	1269.4	548.1

Shanghai Minhang Gymnasium	65	296.4	98.7
Shanghai Songjiang Gymnasium	64	220.3	123.5

Table S5. Queuing time at the actual vaccination clinic

Vaccination clinic	objective	Average queuing time (s)	Average queuing time	Queuing time except preview step
Changshuo Street	45	806	13min26s	5min21s
Deqing	25	845	14min5s	6min12s
Fuxi Street	22	685	11min25s	3min42s
Hefu Town	43	533	8min53s	7min26s
Linghu	54	489	8min9s	6min7s
Longshan Street	16	783	13min3s	3min56s
Shanghai Minhang Gymnasium	65	4809	80min9s	9min2s
Shanghai Songjiang Gymnasium	64	263	4min23s	4min23s
Taihu Street	22	938	15min38s	3min30s
Wukang Street	22	2726	45min26s	20min47s

Table S6. Optimal staffing under the target of maximum number of vaccination recipients

Arrival rate	With preview step [1](Scenario 1)					Without preview step [2](Scenario 2)			
	Vaccination recipients	Preview	Registration	Vaccination	Total staff	Vaccination recipients	Registration	Vaccination	Total staff
55	37	1	1	2	4	42	1	2	3
83	61	1	1	2	4	72	1	2	3
105	104	1	1	2	4	94	1	2	3
155	154	1	2	2	5	149	2	2	4
209	205	2	2	2	6	209	2	2	4
253	245	2	2	3	7	224	2	3	5
309	301	2	2	4	8	301	2	3	5
353	357	3	2	4	9	320	2	4	6
405	370	3	2	5	10	392	2	4	6
460	463	3	2	5	10	457	2	4	6
503	493	3	3	5	11	465	3	5	8
752	737	4	3	5	13	720	3	6	9
1009	945	5	4	6	15	947	4	7	11
1253	1167	5	4	6	15	1177	4	8	12

1509	1352	7	5	7	19	1393	5	9	14
1750	1518	7	6	8	21	1582	6	10	16
2022	2073	7	6	8	21	2067	6	11	17
2524	2596	8	7	10	25	2561	7	13	20
3007	3077	9	8	13	30	3044	8	16	24
3532	3560	10	9	16	35	3596	9	18	27
4041	4096	11	10	18	39	4067	10	20	30
4540	4546	12	11	23	46	4551	11	23	34
5101	5043	13	12	26	51	5087	12	25	37

[1] With preview step: the vaccination clinic was equipped with preview, registration and vaccination steps.[2] Without preview step: the vaccination clinic was equipped with two steps, while the preview and registration steps were combined.

Table S7. Optimal staffing under the target of minimum number of staff & highest efficiency.

Arrival rate	Minimum staff number									Highest efficiency								
	With preview step [1](Scenario 3)					Without preview step [2](Scenario 4)				With preview step [1](Scenario 5)					Without preview step [2](Scenario 6)			
	Vaccinated	Preview	Registration	Vaccination	Total staff	Vaccinated	Registration	Vaccination	Total staff	Vaccinated	Preview	Registration	Vaccination	Total staff	Vaccinated	Registration	Vaccination	Total staff
55	31	1	1	1	3	42	1	1	2	31	1	1	1	3	42	1	1	2
83	60	1	1	1	3	67	1	1	2	60	1	1	1	3	67	1	1	2
105	104	1	1	1	3	94	1	1	2	104	1	1	1	3	94	1	1	2
155	130	1	1	1	3	133	1	1	2	134	1	1	1	3	133	1	1	2
209	184	1	1	1	3	190	1	1	2	160	1	1	2	4	190	1	1	2
253	242	1	2	2	5	224	1	2	3	242	2	2	2	6	224	2	2	4
309	298	2	2	2	6	289	2	2	4	189	2	2	2	6	301	2	2	4
353	333	2	2	2	6	306	2	2	4	333	2	2	3	7	306	2	2	4
405	360	2	2	3	7	351	2	3	5	206	2	2	3	7	351	2	2	4
460	405	2	2	3	7	418	2	3	5	405	2	2	3	7	418	2	3	5
503	498	3	3	4	10	465	3	4	7	498	3	3	4	10	465	3	4	7
752	704	3	4	5	12	720	3	5	8	716	3	4	5	12	720	4	5	9
1009	920	4	4	6	14	936	4	6	10	935	4	4	6	14	936	4	6	10
1253	1109	4	5	7	16	1105	4	7	11	1107	4	5	6	15	1105	5	6	11
1509	1315	5	5	8	18	1360	5	8	13	1315	5	5	8	18	1360	5	7	12
1750	1503	5	5	9	19	1523	6	9	15	1505	5	5	9	19	1532	5	8	13
2022	1978	6	6	10	22	2046	7	10	17	1978	6	6	10	22	2046	6	9	15
2524	2443	7	7	13	27	2527	8	13	21	2599	7	7	12	26	2527	7	11	18
3007	2901	8	8	16	32	2929	8	15	23	2901	9	8	14	31	2911	8	13	21
3532	3460	9	9	18	36	3458	8	17	25	3460	10	9	16	35	3458	9	15	24
4041	3932	10	10	20	40	3914	9	19	28	3990	11	10	18	39	3914	10	17	27
4540	4422	11	11	21	43	4461	10	21	31	4455	12	11	20	43	4461	11	19	30
5101	4923	12	12	23	47	4911	11	22	33	4923	13	12	22	47	4911	12	21	33

[1] With preview step: the vaccination clinic was equipped with preview, registration and vaccination steps.[2] Without preview step: the vaccination clinic was equipped with two steps, while the preview and registration steps were combined.

Table S8. Optimal staffing under the target of least queuing time & shortest queuing number

Arrival rate	Minimum queuing time									Minimum queuing number								
	With preview step [1](Scenario 7)					Without preview step [2](Scenario 8)				With preview step [1](Scenario 9)					Without preview step [2](Scenario 10)			
	Queuing time	Preview	Registration	Vaccination	Total staff	Queuing time	Registration	Vaccination	Total staff	Queuing number	Preview	Registration	Vaccination	Total staff	Queuing number	Registration	Vaccination	Total staff
55	0	1	1	1	3	0	1	1	2	0	1	1	1	3	0	1	1	2
83	0.48	1	1	1	3	0	1	1	2	0	1	1	1	3	0	1	1	2
105	0	1	1	2	4	0	1	2	3	0	1	1	2	4	0	1	2	3
155	0	1	1	3	5	0	1	3	4	0	1	1	3	5	0	1	3	4
209	0	2	2	4	8	0	2	4	6	0	2	2	4	8	0	2	4	6
253	0.24	2	2	5	9	0.01	2	5	7	0	2	2	5	9	0	2	5	7
309	0.12	2	2	6	10	0.01	2	6	8	0	2	2	6	10	0	2	6	8
353	0.24	2	2	7	11	0.09	2	7	9	0	2	2	7	11	0	2	7	9
405	0.72	3	3	7	13	0.02	3	7	10	1	2	3	7	12	0	2	7	9
460	1.2	3	3	7	13	0.01	3	7	10	1	3	3	7	13	0	3	7	10
503	0	3	3	8	14	0	3	8	11	0	3	3	8	14	0	3	8	11
752	0.06	3	4	9	16	0.01	4	9	13	0	3	4	9	16	0	4	9	13
1009	0.24	4	4	10	18	0.06	4	10	14	0	4	4	10	18	0	4	10	14
1253	1.38	4	5	11	20	0.06	5	11	16	3	4	5	11	20	0	5	11	16
1509	5.34	5	5	12	22	0.27	5	12	17	16	5	5	12	22	1	5	12	17
1750	15.54	5	6	13	24	1.95	6	13	19	25	6	6	13	25	7	6	13	19
2022	0	6	7	14	27	0	7	14	21	0	7	7	14	28	0	7	14	21
2524	0	7	8	16	31	0.09	8	16	24	0	8	8	16	32	0	8	16	24
3007	0.24	8	10	18	36	0.42	10	18	28	1	9	10	18	37	3	9	18	27
3532	5.46	11	12	19	42	4.96	12	19	31	30	11	12	19	42	12	11	19	30
4041	11.64	12	14	21	47	9.88	14	21	35	57	12	14	21	47	14	12	21	33
4540	18.9	14	15	23	52	11.18	15	23	38	60	14	15	23	52	20	14	23	37
5101	25.5	15	16	25	56	13.85	16	25	41	81	15	16	25	56	25	15	25	40

[1] With preview step: the vaccination clinic was equipped with preview, registration and vaccination steps.[2] Without preview step: the vaccination clinic was equipped with two steps, while the preview and registration steps were combined.

Table S9. Service utilization for optimal staffing for each purpose.

Arrival rate	With preview step [1]						Without preview step [2]					
	Maximum vaccination recipients		Minimum staff number		Highest efficiency		Maximum vaccination recipients		Minimum staff number		Highest efficiency	
	Vaccination recipients	Utilization	Vaccination recipients	Utilization	Vaccination recipients	Utilization	Vaccination recipients	Utilization	Vaccination recipients	Utilization	Vaccination recipients	Utilization
55	37	0.68	31	0.57	31	0.57	42	0.77	42	0.77	42	0.77
83	61	0.74	60	0.73	60	0.73	72	0.87	67	0.81	67	0.81
105	104	1	104	1	104	1	94	0.9	94	0.9	94	0.9
155	154	1	130	0.84	134	0.87	149	0.97	133	0.86	133	0.86
209	205	0.99	184	0.88	160	0.77	209	1	190	0.91	190	0.91
253	245	0.97	242	0.96	242	0.96	224	0.89	224	0.89	224	0.89
309	301	0.98	298	0.97	280	0.91	301	0.98	289	0.94	301	0.98
353	357	1.02	333	0.95	333	0.95	320	0.91	306	0.87	306	0.87
405	370	0.92	360	0.89	400	0.99	392	0.97	351	0.87	351	0.87
460	463	1.01	405	0.88	405	0.88	457	1	418	0.91	418	0.91
503	493	0.98	498	0.99	498	0.99	465	0.93	465	0.93	465	0.93
752	737	0.98	704	0.94	716	0.96	720	0.96	720	0.96	720	0.96
1009	945	0.94	920	0.92	935	0.93	947	0.94	936	0.93	936	0.93

1253	1167	0.94	1109	0.89	1107	0.89	1177	0.94	1105	0.89	1105	0.89
1509	1352	0.9	1315	0.88	1315	0.88	1393	0.93	1360	0.91	1360	0.91
1750	1518	0.87	1503	0.86	1505	0.86	1582	0.91	1523	0.87	1532	0.88
2022	2073	1.03	1978	0.98	1978	0.98	2067	1.03	2046	1.02	2046	1.02
2524	2596	1.03	2443	0.97	2599	1.03	2561	1.02	2527	1.01	2527	1.01
3007	3077	1.03	2901	0.97	2901	0.97	3044	1.02	2929	0.98	2911	0.97
3532	3560	1.01	3460	0.98	3460	0.98	3596	1.02	3458	0.98	3458	0.98
4041	4096	1.02	3932	0.98	3990	0.99	4067	1.01	3914	0.97	3914	0.97
4540	4546	1.01	4422	0.98	4455	0.99	4551	1.01	4461	0.99	4461	0.99
5101	5043	0.99	4923	0.97	4923	0.97	5087	1	4911	0.97	4911	0.97

[1] With preview step: the vaccination clinic was equipped with preview, registration and vaccination steps.[2] Without preview step: the vaccination clinic was equipped with two steps, while the preview and registration steps were combined.

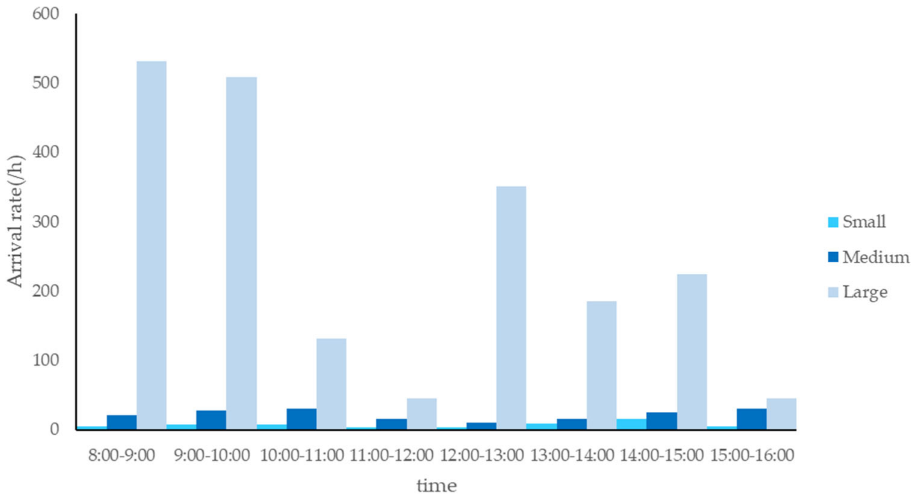

Figure S1. Arrival rate of representative clinics

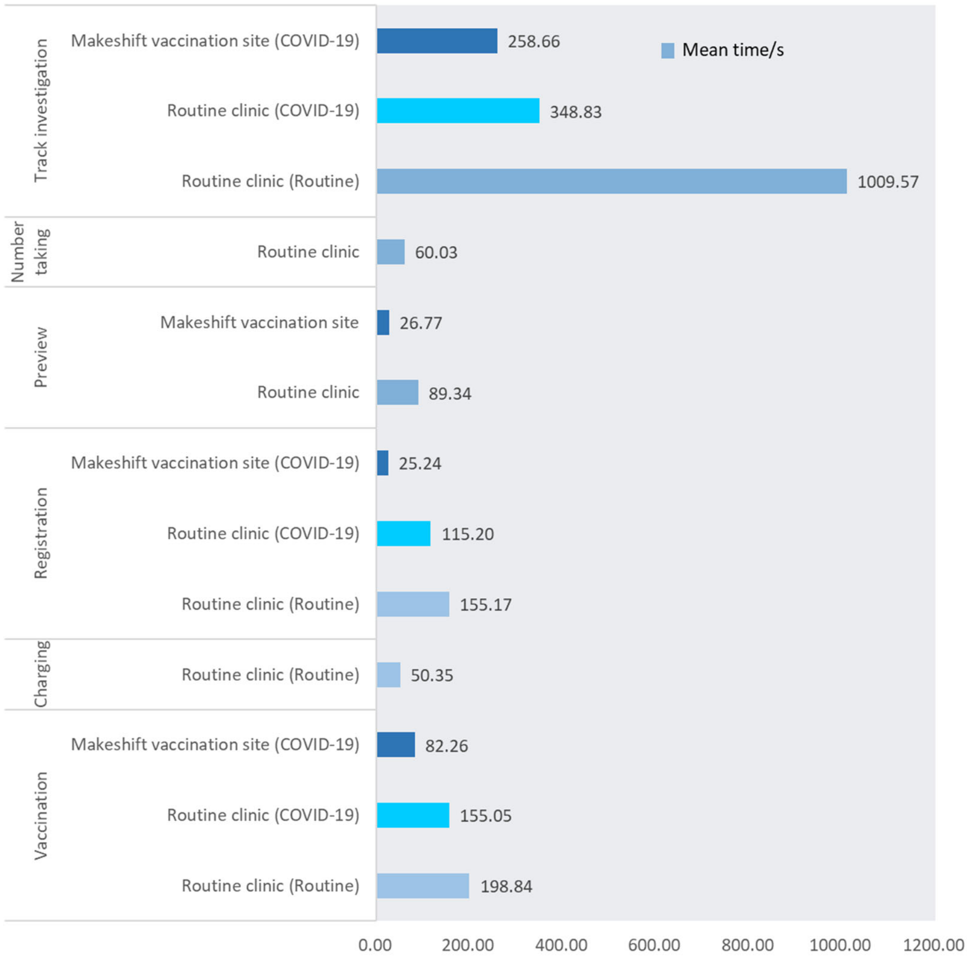

Figure S2. Time spent in each step in each clinic

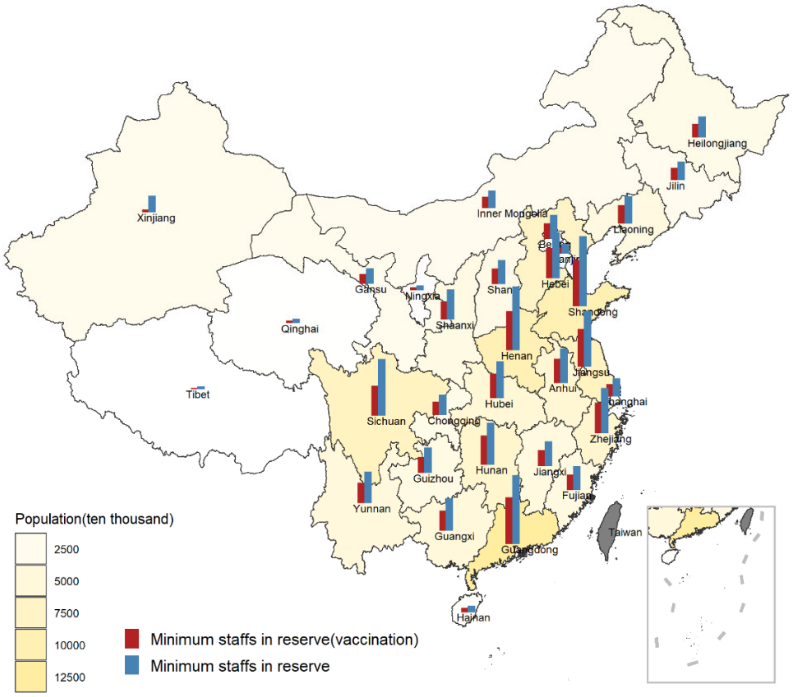

Figure S3. Distribution of the number of population and staffs by province. The yellow area represented the resident population in each province, with darker colors indicating a larger population. The blue and red bars indicated the total number of staff currently available as vaccination clinic staff and the total number of vaccination step staff in each province, respectively, with higher bars representing larger numbers.

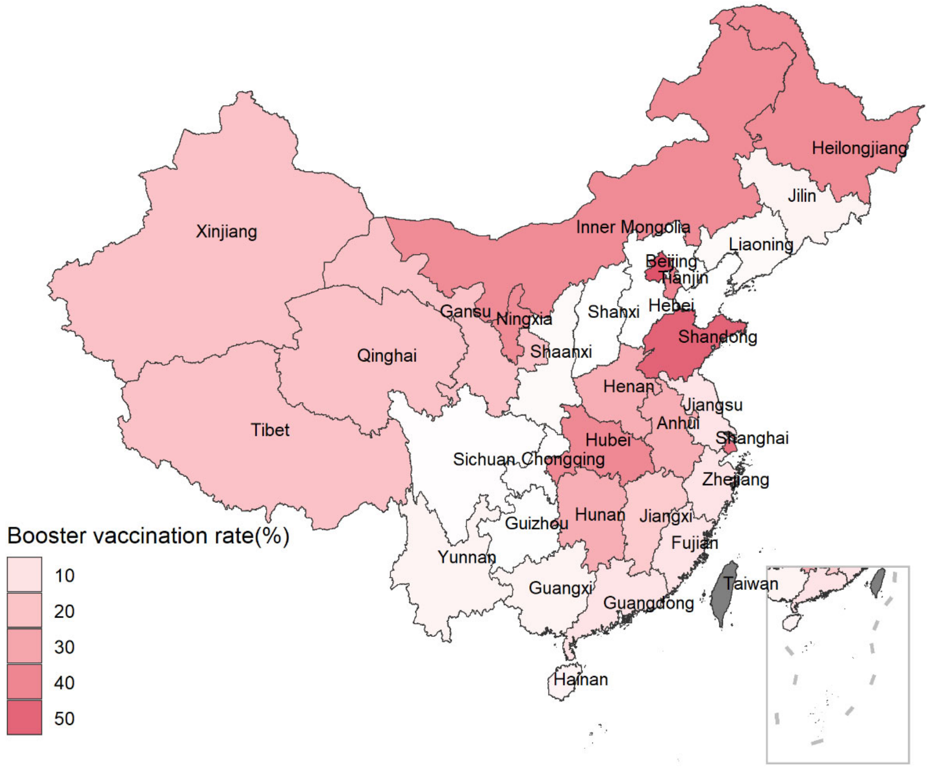

Figure S4. Booster vaccination rate by province**.** The red area indicated the vaccination rate of booster shots in each province; the darker the color, the higher the vaccination rate.

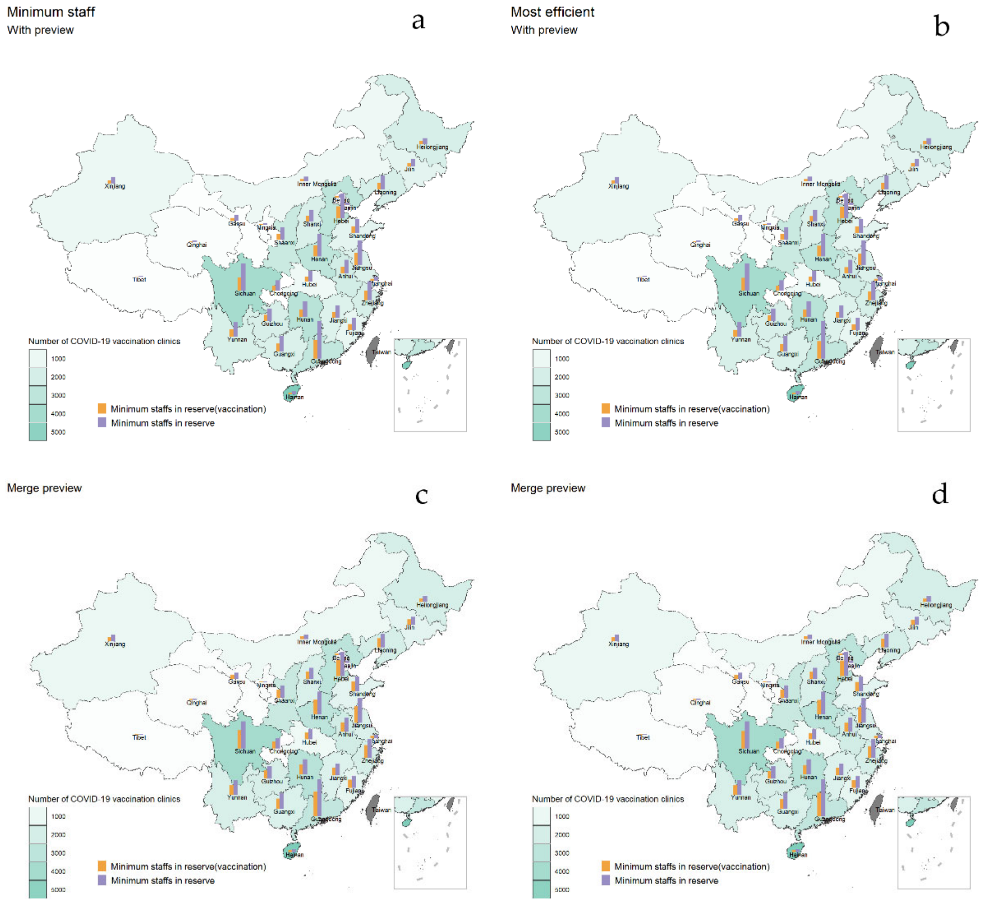

Figure S5. Optimal staffing to achieve the goals within 6 months. The green area in the map represented the number of COVID-19 vaccination clinics in each province, the darker the color the more COVID-19 vaccination clinics. The orange bar represented the minimum number of staff needed to be stockpiled for the vaccination step at the vaccination clinics optimized by the simulation model under the goal of raising the booster vaccination rate to the full immunization level in 3 months in different scenarios. The higher the bar, the more people needed to be stocked. The purple bar represented the minimum number of staff to be stocked in the vaccination clinic, and the higher the bar, the higher the number of staff to be stocked. (**a**,**b**) Optimization when the vaccination clinic was equipped with preview, registration and vaccination steps; (**c**,**d**) Optimization when the preview and registration steps were combined.

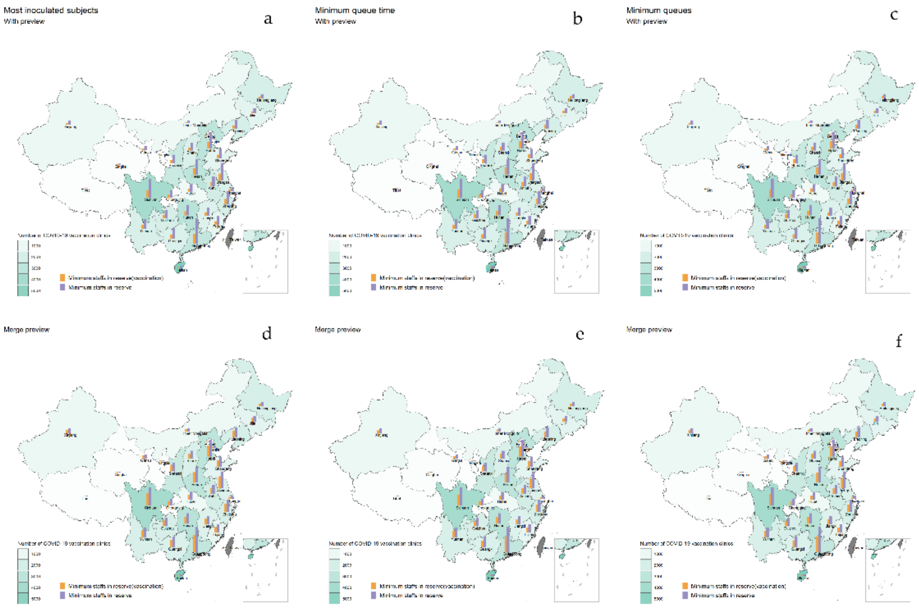

Figure S6. Optimal staffing to achieve the goals within 3 months when the epidemic was stable. The green area in the map represented the number of COVID-19 vaccination clinics in each province, the darker the color the more COVID-19 vaccination clinics. The orange bar represented the minimum number of staff needed to be stockpiled for the vaccination step at the vaccination clinics optimized by the simulation model under the goal of raising the booster vaccination rate to the full immunization level in 3 months in different scenarios. The higher the bar, the more people needed to be stocked. The purple bar represented the minimum number of staff to be stocked in the vaccination clinic, and the higher the bar, the higher the number of staff to be stocked. (**a,b,c**) Optimization when the vaccination clinic was equipped with preview, registration and vaccination steps; (**d,e,f**) Optimization when the preview and registration steps were combined.
